# The Prevalence and Distribution of Spina Bifida in a Single Major Referral Center in Malaysia

**DOI:** 10.3389/fped.2017.00237

**Published:** 2017-11-09

**Authors:** Adibah Sahmat, Renuka Gunasekaran, Siti W. Mohd-Zin, Lohis Balachandran, Meow-Keong Thong, Julia P. Engkasan, Dharmendra Ganesan, Zaliha Omar, Abu Bakar Azizi, Azlina Ahmad-Annuar, Noraishah M. Abdul-Aziz

**Affiliations:** ^1^Faculty of Medicine, Department of Parasitology, University of Malaya, Kuala Lumpur, Malaysia; ^2^Faculty of Medicine, Department of Paediatrics, University of Malaya, Kuala Lumpur, Malaysia; ^3^Faculty of Medicine, Department of Rehabilitation Medicine, University of Malaya, Kuala Lumpur, Malaysia; ^4^Faculty of Medicine, Department of Surgery, University of Malaya, Kuala Lumpur, Malaysia; ^5^Rehabilitation Medicine Department, Sunway Medical Centre, Petaling Jaya, Malaysia; ^6^Department of Surgery, National University of Malaysia, Kuala Lumpur, Malaysia; ^7^Faculty of Medicine, Department of Biomedical Science, University of Malaya, Kuala Lumpur, Malaysia

**Keywords:** spina bifida, University Malaya Medical Centre, prevalence, distribution, 13 years study

## Abstract

**Background:**

The aim of this study is to review the medical history of patients with spina bifida, encompassing both aperta and occulta types born between the years 2003 until 2016, spanning a 13-year time period. We assessed each patient and maternal parent information, details of the defects, and conditions associated with the primary defect. We also include information on patients’ ambulation and education level (where available).

**Methods:**

Data from the Department of Patient Information University of Malaya Medical Centre (UMMC), Malaysia was captured from spina bifida patients (ICD10: Q05 spina bifida). Data involved patients referred to UMMC between 2003 and 2016 and/or born in UMMC within that particular time frame. We filtered and extracted the information according to the data of clinical examination, medical review, and social history provided in the medical records.

**Results:**

A total of 86 patient records with spina bifida were analyzed. Spina bifida prevalence rate in this study ranged from 1.87 to 8.9 per 1,000 live births depending on weightage. We note that ethnicity was a factor whereby the highest numbers of spina bifida were from Malays (*n* = 36, 41.86%), followed by equal numbers of Chinese and Indians (*n* = 24, 27.91%). The highest number of diagnoses reported was myelomeningocele type-spina bifida (*n* = 39, 45.35%). The most common site of the spina bifida lesion was located at the lumbar region irrespective of aperta or occulta types (*n* = 23, 26.74%). Data on other associated phenotypes of spina bifida such as hydrocephalus and encephalocele was also captured at 37.21% (*n* = 32) and 1.16% (*n* = 1), respectively. In terms of mobility, 32.84% (*n* = 22/67) of patients between the ages 4 and 16 years old were found to be mobile. As many as 36.07% of patients ranging from 5 to 16 years of age (*n* = 22/61) received formal education ranging from preschool to secondary school.

**Conclusion:**

The prevalence of spina bifida in UMMC is as according to international statistics which is in the range of 0.5–10 per 1,000 live births. Majority of the reported cases were males, Malays, full term babies, and of the myelomeningocele phenotype located at the lumbar region.

## Introduction

Neural tube defects (NTDs) are the most common birth defect of the central nervous system and they occur at a range of 0.5–10 or more in 1,000 live births worldwide (1). NTD is a multifactorial condition resulting from the failure of embryonic neural tube closure. Clinical phenotypes of NTDs depend on the points of embryonic neural tube closure (2), whereby craniorachischisis results in the simultaneous exposure of the brain and the spinal cord. Anencephaly is due to failure of closure at the midbrain and/or forebrain, resulting in an exposed brain (1, 3). Both craniorachischisis and anencephaly are incompatible with postnatal life. Spina bifida is due to failure of closure of the spinal neural tube (1). Majority of spina bifida are non-syndromic NTDs (4). Syndromic spina bifida that is NTD accompanied by other associated disorders may include Jarcho–Levin syndrome (5), X-linked heterotaxy (6), DiGeorge syndrome (7), as well as Turner syndrome (8) as examples of the accompanying genetic problems associated with syndromic spina bifida that accounts for less than 10% of NTDs (4, 9).

**Figure 1:**
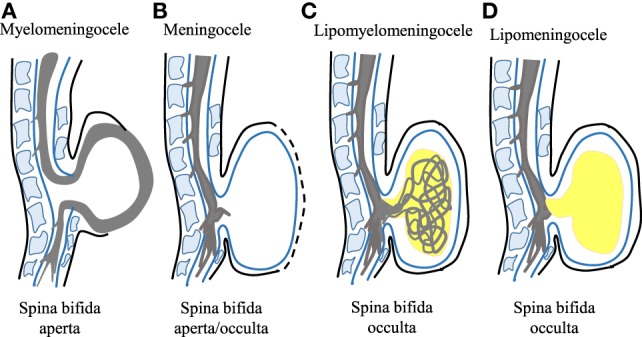
Schematic representation of different spina bifida subphenotypes. **(A)** Myelomeningocele is shown whereby the spinal cord lies outside the spinal canal. This phenotype represents the severe form of spina bifida aperta. **(B)** Meningocele is shown whereby the spinal cord does not lie outside the spinal canal. This phenotype represents spina bifida occulta or spina bifida aperta depending on the presence or absence of neural matter in its herniated sac. **(C)** Lipomyelomeningocele that is the spina bifida occulta type is shown with the presence of intermeshed lipid globules (in yellow) and spinal cord. **(D)** Lipomeningocele that represents spina bifida occulta is shown mimicking the meningocele but with the presence of lipid globules.

Of all the types of NTDs, spina bifida is known to be the most common type and spina bifida patients have a higher chance of survival (10). It is caused by the failure of the spinal neural tube to close at approximately day 28 of human gestation (11). Spina bifida can appear in two forms; spina bifida occulta and spina bifida aperta. Spina bifida occulta is a closed form of spina bifida where the lesion is covered with skin and the spinal cord is not exposed (4). Meanwhile, spina bifida aperta occurs when the spinal cord is exposed to its surrounding environment with or without a herniating sac, and without skin covering (9, 11, 12). The array of phenotypes of spina bifida can be further divided into a number of subtypes; myelomeningocele, meningocele, lipomyelomeningocele, and lipomeningocele depending on the pathophysiology of the lesion (Figure 1).

The risk factors of spina bifida in Malaysia have never been explored considering that spina bifida is a multifactorial condition (13–16); therefore, environmental factors in Malaysia are a relevant point in the etiology of spina bifida and require further understanding. Hence, basic patients’ and maternal parents’ information, including gender predisposition, ethnicity, birth weight, maternal age, details of the defects, and associated conditions, were included for a clearer scenario of the defect in our cohort. Also, patients’ ambulation and education were scrutinized to improve the management and treatment depending on the severity of defect in each patient. This study focuses on the occurrence and follow-up of spina bifida cases in a major hospital in the capital of Malaysia, Kuala Lumpur from the year 2003 until 2016. This single institutional study is made significant due to the lack of publications on spina bifida in Malaysia.

## Materials and Methods

### Human Ethics Approval

Data were retrieved from the University of Malaya Medical Centre (UMMC), Department of Patient Information after approval from the institutional UMMCEC Human Ethics committee (MEC Ref. No. 914.5).

### Data Collection

University of Malaya Medical Centre is part of University of Malaya and it is a semi-government-funded medical institution situated in Kuala Lumpur, Malaysia. It serves as a referral medical center for the whole of Malaysia (17). Data for this retrospective study were obtained from the UMMC Department of Patient Information. Records obtained were from patients diagnosed with spina bifida according to ICD10: Q05 (Spina bifida) in the period of 13 years (2003–2016). Data captured included (a) demographic details on patient’s ethnicity, gender, year of birth, birth weight, birth term, mother’s age, and mode of delivery (spontaneous vaginal delivery/cesarean); (b) details of defects on diagnosis, open or closed lesion, level of lesion, and syndromic or non-syndromic; (c) presence of other conditions associated with spina bifida such as hydrocephalus including any insertion of the ventriculo-peritoneal (VP) shunt; and (d) patients’ ambulation and education.

### Statistical Analysis

The data were analyzed using the Statistical Program for the Social Sciences (SPSS, version 22.0, 2013, IBM corp). Contingency table was used to display frequency distribution of the ethnicity and genders based on the types of diagnosis and tested using Chi-square. Differences with *p* < 0.05 were considered significant indicating a relationship between the variables. GraphPad Prism 5 was used to generate graphs.

## Results

### Estimated Prevalence Rate of Spina Bifida in Our Cohort

Eighty-six patient records were confirmed as spina bifida according to specific information retrieved from the records after unbiased filtering. From the 86 patients, the estimated number of spina bifida in-house and referred cases annually in UMMC alone is 7. Using 139 as the total number of government hospitals in Malaysia (18) and 520,000 as the annual average number of live births based on the Department of Statistics Malaysia (19), the estimated prevalence rate of spina bifida in our study sample numbers a high of 1.87 per 1,000 live births.

Calculation method 1:

$$\frac{\left(\text{Reported spina bifida case in UMMC per year}\times \text{total no. of government hospital}\right)}{\text{Malaysian average number of live births}}\times 1,000$$

$$=\frac{\left(7\times 139\right)}{520,000}\times 1,000$$

=1.87per1,000live births.

Taking into account that UMMC is a referral center, there exists the potential that UMMC will record higher numbers. However, other issues, which potentially decrease the number of occurrences, will include termination of pregnancies of spina bifida, miscarriage of spina bifida and cases of spina bifida, which go unreported. It is not possible to distinguish spina bifida cases born and referred to UMMC. It is not known which among the 86 live births occurred in UMMC and which were referred. Therefore, weighting adjustment was performed accordingly (20).

We obtained a total of 206 cases from the Department of Patient Information, which was classified ICD10: Q05. However, only 86 cases were confirmed as mentioned above and as a result, as many as 120 cases listed as spina bifida under the ICD10: Q05 were confirmed to be incomplete. Therefore, a second calculation was performed to take this into account. We obtained weighting of 8.9 based on this calculation.

Calculation method 2:

$$\;\text{Weighting adjustment}=\frac{\text{\% stratum of population}}{\text{\% stratum of sample}}$$

$$\text{\% stratum of population}=\frac{206}{100}\times 0.18\times 100=37.08\text{\%}$$

$$\;\text{\% stratum of sample}=\frac{86}{206}\times 100=41.75\text{\%}$$

$$\begin{array}{cc} \;\text{Weighting adjustment} & =\frac{37.08\text{\%}}{41.75\text{\%}}=0.89\\ & =8.9\;\text{per}\;1000\;\text{live birth} \end{array}$$

### Demographic Data Analysis

**Figure 2:**
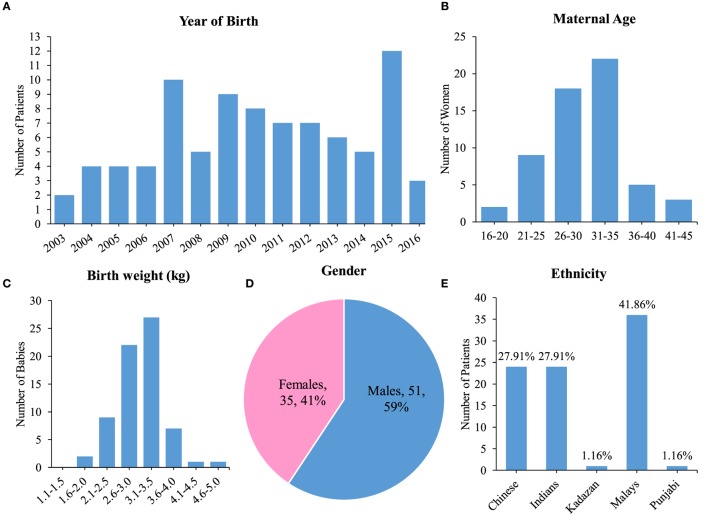
Demographics of spina bifida in the University of Malaya Medical Centre between the years 2003 until 2016. **(A)** Bar chart showing year of birth for patient cohort. **(B)** Maternal age with highest peak at 31–35 years old. **(C)** Birth weight with highest peak at 3.1–3.5 kg. **(D)** Gender preponderance of spina bifida patients. **(E)** Ethnicity of spina bifida patients.

The number of spina bifida cases between 2003 until 2016 is as shown in Figure 2A. A total of 35% (*n* = 30) patients were born through spontaneous vaginal delivery while 42% (*n* = 36) were born by lower segment Cesarean section. The other 20 cases were not accounted for in terms of mode of delivery. The maternal age during childbirth ranged from 17 to 42 years old and the most affected age were below than 35 years old (Figure 2B). The birth weight mostly occurred at 3.1 to 3.5 kg and it ranged between 1.3 and 4.6 kg (Figure 2C). Data for birth term was retrieved from 81% of cases, where 75% were full term babies and 6% were premature babies. Pregnancy age less than 37 weeks were considered as premature while 37 weeks and above as full term. In our cohort, 59% were males and 41% females (Figure 2D). Majority of cases in the database were of Malay ethnicity (41.86%; *n* = 36), followed by an equal number of Chinese and Indians at 27.91% (*n* = 24 each). There was only a single case each of ethnic minorities, a Kadazan and a Punjabi child, which registered at 1.16% (*n* = 1 each) (Figure 2E).

### Types of Defects

The most commonly reported NTD type was spina bifida with myelomeningocele (45.35%, *n* = 39) (Table 1). There was a single case each of encephalocele with meningocele (1.16%) and 11 cases of lipomyelomeningocele (12.79%) (Table 1). The most commonly reported level of spina bifida lesion was at the lumbar region (26.7%, *n* = 23) (Table 2). Non-syndromic spina bifida represented the majority of the cases (91%). In this study, 37% (*n* = 32) of spina bifida patients also had hydrocephalus, which is considered an associated NTD, 40% (*n* = 34) were noted to be without hydrocephalus and there were no specific records for 23% of the cases. Surgery to insert VP shunts had to be performed for 97% of patients with hydrocephalus.

**Table 1:** Number and percentage of patients with types of spina bifida recorded.

Diagnosis	Number of patients	Percentage
Spina bifida only	12	13.95
Myelomeningocele	39	45.35
Meningocele	13	15.12
Encephalocele with meningocele	1	1.16
Lipomyelomeningocele	11	12.79
Lipomeningocele	10	11.63
Total	86	100

**Table 2:** Number and percentages of patients with spina bifida and level of lesion.

Level of lesion	Number of patients	Percentage
Thoracic	3	3.49
Thoracolumbar	6	6.98
Lumbar	23	26.74
Lumbosacral	18	20.93
Sacral	16	18.60
Sacrococcygeal	1	1.16
Not available	19	22.09
Total	86	100

### Mobility and Education

Out of the 86 patients in our cohort, 22 over 67 (32.84%) of patients between age 4 and 16 years old were captured in terms of mobility where they were able to ambulate independently using aids without having to depend on others. The rest of the data were not captured. We also discovered 22 out of 61 patients (36.07%) range from 5 to 16 years old have varying levels of education ranging from playschool to secondary school. Two patients (3.28%) age 7 and 14 years old were not attending school. The other 60.66% (*n* = 37) patients were unaccounted for in terms of education.

### Analysis of Diagnosis

**Figure 3:**
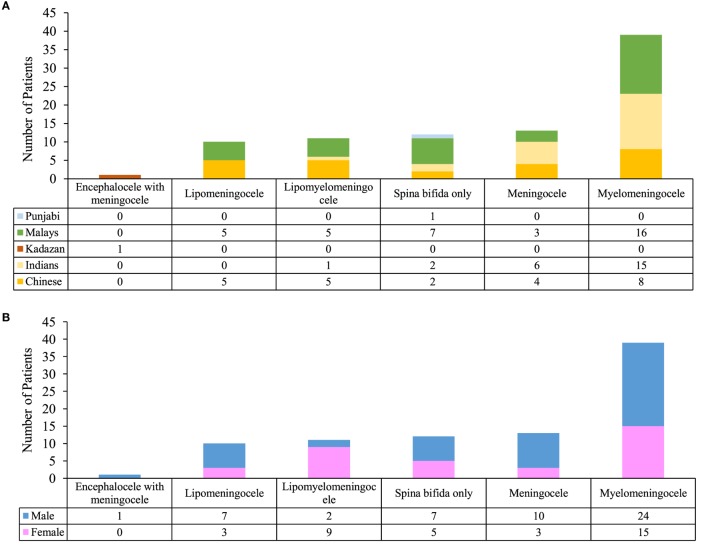
Analysis of diagnosis. **(A)** Comparison between ethnicity and type of diagnosis. **(B)** Comparison between genders and type of diagnosis.

There was no significant relationship between the genders in comparison to the types of diagnosis. However, there was an association between ethnicity and the types of diagnosis (*p* < 0.05) (Figure 3).

## Discussion

This study aims to carefully navigate data obtained from patient records by unbiased filtering, followed by extrapolation of data to produce coherent and potentially revealing information which can be used fruitfully for the betterment of the quality of life of spina bifida patients. Our study revealed the rate of prevalence of spina bifida in a major referral center in Kuala Lumpur, type of spina bifida, leading ethnic group, maternal age, method of delivery, birth weight, birth term, gender, incidence of hydrocephalus and VP shunt insertion, types of defect, and the level of lesion, including mobility and the level of education.

It is the nature of the retrospective study to extract data from records and run analysis on the available data. However, data retrieved were incomplete due to (a) information and clinical examinations provided by physician in the records are based on the urgency in attending treatments or procedure instead of diagnosis or cause of spina bifida, (b) the 13 years’ records of patients referred to UMMC covers only the period of time the patients were admitted at a later age and so, information regarding him or her during birth is unknown, and (c) discrepancy of patients in providing sufficient information to their physician. Nevertheless, the discussions will be based on the captured data from our records and any discrepancy has been mentioned and accounted for. Hence, we suggest a more standardized form of tabulating information for patient records, including validation of imaging. Also, online links should be made from the patients’ records to the medical imaging repository to ensure the information can be verified.

### Prevalence of Spina Bifida in UMMC As an Indicator for Urban Malaysia

Data captured from 86 patients (Figure 2A) revealed prevalence rate of spina bifida in the present study ranged from 1.87 to 8.9 per 1,000 live births that reveals a much higher prevalence rate than that which was previously recorded. The result obtained was unsurprisingly high owing the fact that the data were retrieved from UMMC, which serve as a major referral hospital in Malaysia (17). As far as detailed examination and treatment are concerned, most spina bifida patients will be regionalized to the referral hospital where health care from specialists are provided with the advancement of medical treatment and investigations (4, 21). A prospective cohort study of neonates with spina bifida using the data from the Malaysian National Neonatal Registry has shown a prevalence rate of 0.11 per live 1,000 births (22). Data captured from that study include patients born in 2009 from 32 Malaysian hospitals. In another study of a small population in the Kinta district, Malaysia reported an incidence of NTDs at 0.73 per 1,000 births but does not specify the NTD phenotype (23). The EUROCAT (European Surveillance of Congenital Anomalies) estimates the rate of spina bifida in Europe at 0.51 per 1,000 live births from year 2003 to 2007 (4, 24). The frequency is found to be higher in the United States of America and United Kingdom (9, 25, 26). Meanwhile, certain region in China such as Shanxi Province has a much higher preponderance to this condition than the other parts of the world (13, 27). Since the Boo et al.’s (22) study, there have not been any other studies on spina bifida in Malaysia. Therefore, our study aimed at garnering more current data on the state of spina bifida in Malaysia. Result from this study revealed the prevalence rate of spina bifida to be similar according to that cited internationally which is 0.5–10 per 1,000 live births for NTD cases (1).

### Distribution of Spina Bifida

Our data conform to the global scenario of myelomeningocele, reported as the most common and severe form of spina bifida (4) (Table 1). Myelomeningocele is commonly associated with hydrocephalus and encephalocele (4). Thus, surgical intervention is required for myelomeningocele patients to cover the exposed spinal cord in order to prevent infection and insertion of VP shunt to treat hydrocephalus where necessary (28). Lesions occurred mostly at the lumbar region (Table 2) as previously been reported by “The Spina Bifida Research Resource” (29). These data tally with the United States of America.

In addition, syndromic spina bifida was reported in 9% of the total number of patients. The represented phenotypes include autism and 48XY (intra-abdominal gonads). Unfortunately, information about karyotype analysis is limited as they were not provided in the medical records to confirm the diagnosis. Data on antenatal ultrasound to detect spina bifida was also not available in the present study. Ultrasound examination during prenatal check-ups is used in the early detection of spina bifida (30). However, it is not always accurate and sometimes failed to diagnose the spina bifida especially the occulta type (31).

### Maternal Health Influence Spina Bifida Neonates

Our records show that maternal age during childbirth mostly affected at age below 35 years old (Figure 2B). This finding maybe quite revealing in that the typical childbearing age in Malaysia is between 20 and 35 years old (32, 33), so it would make sense for the highest number to be in that particular age range. Nevertheless, the presented data suggests that healthy mothers at their ideal childbearing age are also affected and this may be due to genetic or environmental triggers (34). One caveat of this study is the lack of information about folic acid intake by the mothers. Although the Ministry of Health Malaysia recommends periconceptional folic acid supplementation to all pregnant women to promote healthy pregnancy (35), the intake of folic acid is not mandatory, thus it may give rise to a higher risk of NTD.

Our data show that full term babies born with spina bifida were within the normal birth weight (Figure 2C) as indicated by the Pediatric and Pregnancy Nutritional Surveillance System, Center for Disease Control and Prevention (CDC). There have been other studies that suggest low birth weights were greater in NTD offspring without specifying the phenotype (15, 36). According to the CDC, newborns should weigh more than 2.5 kg and less than 4 kg (37).

As for gender, male patients hold more than half of the overall percentage, which was 59% (Figure 2D). The number of males was also higher in majority of the spina bifida subphenotypes (Figure 3B). The discrepancy might be related to the geographical factor where males dominate the general Malaysian population. In the year 2014–2016, Malaysia has 0.9 million more males compared to females (19). However, this result contradicts studies from other countries stating that females are more predisposed to NTDs compared to males (38, 39). Recent reports from Bangladesh also record a higher preponderance of spina bifida among males (38, 40). The United Kingdom population-based study found the number of females is lesser than males in overall risk of congenital anomalies. Regardless of the phenomenon, this pattern appears to be reversed in NTD cases where females have a higher risk of NTDs at birth (41).

From our findings, Malay patients records the highest number of spina bifida cases (Figure 2E) particularly in myelomeningocele and spina bifida only subtypes (Figure 3A). In the previous records by the National Birth registry, NTDs were highest among the Sarawak indigenous people and lowest among the Chinese (22).

### Education, Mobility, and the Issue of Management of Spina Bifida in Malaysia

Our data show that a proportion of our spina bifida patients pursued education (36.07%, *n* = 22/61 of patients ranging from 5 to 16 years old). Most of them are capable of enrolling in the national curriculum and participate in the process of learning. Despite that, two patients were not attending school due to unknown reason and more than half of the number of patients were unaccounted for in terms education. Studies of Malaysian school-aged children with physical disability, including spina bifida identified numerous restrictions to achieve education that ranges from which difficulties in managing urinary or bowel incontinence, dependent mobility, inaccessible school facilities, and societal or environmental barrier (42, 43). Mobility which was achieved by 32.84% (*n* = 22/67) of patients ranging from 4 to 16 years old aided by wheelchairs, crutches, and ankle-foot orthosis are important to retain patients’ mobility (44). There is correlation between mobility and the level of lesion. Higher level of lesion leads to more mobility difficulties, such as dependant ambulation, imbalance, and use of mobility aids compared to patients with lower level of lesion (42). Besides that, most of spina bifida patients are diagnosed with neurogenic bladder dysfunction and they require a proper bladder management (45). Although we lack data on the bladder management among our patients, the use of clean intermittent catheterization is mainly utilized in the prevention of kidney damage (46). It is noteworthy that different spina bifida patient needs different treatment and management as it depends on the level of lesion and the type of diagnosis. Front liners among the medical community for example emergency room doctors and general practitioners as well obstetrics and gynecologists should be continuously educated in the management of spina bifida as it is a common condition. Then only can patients and parents be trained as early as possible so that the patients environment can be inclusive and that they are able to eventually live independently (47).

### Summary

Our data show that the prevalence of spina bifida is higher compared to previously published records. Based on our data, we found that certain well-accepted norms such as myelomeningocele, lesion at lumbar region, and higher occurrence of non-syndromic spina bifida compared to syndromic spina bifida are applicable to the Malaysian urban scenario. We urge a closer and deeper understanding of the etiology of spina bifida and suggest that the UMMC cohort may be useful for the understanding of spina bifida. More studies involving the latest occurrence of spina bifida covering the entire Malaysia are necessary and paramount to further understand this common central nervous system malformation.

## Ethics Statement

This study was carried out in accordance with the recommendations of UMMC Medical Research Ethics Committee (MREC) with written informed consent from all subjects. All subjects gave written informed consent in accordance with the Declaration of Helsinki. The protocol was approved by the UMMC Medical Research Ethics Committee (MREC) (Ethics no: MEC Ref. No. 914.5).

## Author Contributions

AS and SWM-Z conceived and designed the experiments, performed the experiments, analyzed the data, wrote the manuscript, and prepared figures and/or tables. RG analyzed the data, performed statistical analysis, wrote the manuscript, and prepared figures and/or tables. LB performed the experiments, analyzed the data, wrote the manuscript, and prepared figures and/or tables. TM-K conceived and designed the experiments, contributed reagents/materials/analysis tools reviewed drafts of the manuscript. JE and ZO contributed reagents/materials/analysis tools and reviewed drafts of the manuscript. DG and AA-B conceived and designed the experiments and reviewed drafts of the manuscript. AA-A conceived and designed the experiments, analyzed the data, and reviewed drafts of the manuscript. NMA-A conceived and designed the experiments, analyzed the data, contributed reagents/materials/analysis tools, reviewed drafts of the manuscript, and wrote the manuscript.

## Conflict of Interest Statement

The authors declare that the research was conducted in the absence of any commercial or financial relationships that could be construed as a potential conflict of interest.
